# Inhibition of Cerebral Ischemia/Reperfusion Injury by MSCs-Derived Small Extracellular Vesicles in Rodent Models: A Systematic Review and Meta-Analysis

**DOI:** 10.1155/2022/3933252

**Published:** 2022-10-06

**Authors:** Lei Zhang, Chaoying Pei, Dan Hou, Guoshuai Yang, Dan Yu

**Affiliations:** Department of Neurology, Affiliated Haikou Hospital of Xiangya School of Medicine, Central South University, Haikou 570208, China

## Abstract

Small extracellular vesicles (sEVs) secreted by mesenchymal stem cells (MSCs) have shown great therapeutic potential in cerebral ischemia-reperfusion injury (CIRI). In this study, we firstly performed a systematic review to evaluate the efficacy of MSCs-derived sEV for experimental cerebral ischemia/reperfusion injury. 24 studies were identified by searching 8 databases from January 2012 to August 2022. The methodological quality was assessed by using the SYRCLE ‘s risk of bias tool for animal studies. All the data were analyzed using RevMan 5.3 software. As a result, the score of study quality ranged from 3 to 9 in a total of ten points. Meta-analyses showed that MSCs-derived sEVs could effectively alleviate neurological impairment scores, reduced the volume of cerebral infarction and brain water content, and attenuated neuronal apoptosis. Additionally, the possible mechanisms of MSCs-derived sEVs for attenuating neuronal apoptosis were inhibiting microglia-mediated neuroinflammation. Thus, MSCs-derived sEVs might be regarded as a novel insight for cerebral ischemic stroke. However, further mechanistic studies, therapeutic safety, and clinical trials are required. Systematic review registration. PROSPERO CRD42022312227.

## 1. Introduction

Stroke has the characteristics of high economic burden, high incidence, high recurrence rate, high mortality rate, and high disability rate, among which the incidence of ischemic stroke (IS) is the highest [1]. Treatment of acute ischemic stroke (AIS) is based on the timely restoration of blood flow to the ischemic brain tissue by intravenous thrombolysis (IVT) and/or mechanical thrombectomy (MT) [2]. However, recanalization may aggravate the neurological deficit after cerebral ischemia, that is, cerebral ischemia-reperfusion injury (CIRI).

CIRI refers to the phenomenon that ischemic injury of the brain leads to the injury of brain cells, which is further aggravated after the recovery of blood reperfusion [3]. The specific mechanism may be related to oxygen free radicals through lipid peroxidation, protein degeneration, mitochondrial apoptosis, and activation of death receptors during reperfusion [3]. This kind of injury can further lead to aggravation of brain injury and neurological dysfunction, and even nerve cell death [4]. At present, CIRI has attracted increasing attention. However, neuroprotective drugs used to treat the neuronal injury caused by CIRI have limited therapeutic effects. It is of great significance to develop a more effective new approach for the treatment of CIRI.

Mesenchymal stem cells (MSCs) possess the characteristics of immunoregulation, multidirectional differentiation potential, easy access, rapid proliferation in vitro, low activity loss after cryopreservation, low immunogenicity, and nontoxic side effects [5]. Several previous studies have demonstrated tremendous potential of MSCs in treating CIRI [6], myocardial ischemia-reperfusion injury (IRI) [7], hepatic IRI [8], intestinal IRI [9], renal IRI [10], lung IRI [11], retinal IRI [12], and spinal cord IRI [13] exhibiting specific mechanisms of action, such as angiogenesis, antiapoptosis, anti-inflammation, and tissue regeneration. In recent years, the literature supports that the paracrine mechanism of MSCs is mediated at least in part by extracellular vesicles (EVs) [14].

Extracellular vesicles (EVs) are membrane vesicles that are released into the surrounding extracellular environment and can be divided into the subgroups of microvesicles and exosomes [15, 16]. Exosomes are vesicles released by a cell that is between 30 and 100 nm in diameter, which is composed of a multiprotein complex, containing receptors, enzymes, transcription factors, extracellular matrix (ECM) proteins, nucleic acids (mtDNA, ssDNA, dsDNA, mRNA, and miRNA), and also lipids [17–19]. Previous studies have reported that exosomes showed similar or equivalent therapeutic function to MSCs to reduce injury caused by ischemia/reperfusion in a variety of tissues and organs, including the spinal cord [20], kidney [21], liver [22], heart [23], lung [24], brain [25–49], and intestine [50]. Treatment with exosomes overcomes the limitations associated with cell-based therapies and offers several advantages such as easy entry into the ischemic brain after their administration owning to their lipophilicity, less or no immunogenicity and tumorigenicity, and less incidence of occlusion in the microvasculature [27]. Due to difficulties existing in the isolation of a pure population of exosomes through the method used in present studies, we will use the term “small extracellular vesicles” (sEVs) to refer to EVs less than 200 nm in diameter, according to the updated guidelines of the International Society for Extracellular Vesicles of 2018 (MISEV2018) [51].

In this study, 24 published literatures [26–49] were systematically reviewed and meta-analyzed to evaluate the safety and efficacy of exocrine derived from mesenchymal stem cells in the treatment of CIRI. Thus, it can provide a reference basis for the clinical use of exocrine derived from mesenchymal stem cells in the future treatment of CIRI, promote the study of more and larger-scale exocrine derived from mesenchymal stem cells in the treatment of CIRI and put it into clinical applications in a timely manner.

## 2. Methods

### 2.1. Search Strategies

Relevant papers published between January 2012 and August 2022 were screened in PubMed, Web of Science, Embase, Cochrane Library, CNKI (China National Knowledge Infrastructure), VIP Database for Chinese Technical Periodicals, Wanfang Database, and Chinese Biomedical Literature Database. The following keywords were used for literature retrieval: “exosomes”, “extracellular vesicles”, “EVs”, “Reperfusion Injury”, “MSCs”, and “Mesenchymal Stem Cells.” All searches used combinations of keywords and free words, while appropriate adjustments were made according to the corresponding database. The relevant systematic reviews and references cited in the searched articles were also filtered to avoid leaving out any potentially usable studies. Besides, other related articles were also available by examining the reference list by hand. There is no restriction on publication language or publication status. Take PubMed as an example, the specific retrieval strategies are shown in the Supplementary 2 File.

### 2.2. Inclusion and Exclusion Criteria

Studies meeting the following criteria at the same time were included in this paper: (1) animal model: rats or mice of any age or gender exposed to cerebral ischemia-reperfusion injury; (2) intervention: sEVs derived from mesenchymal stem cells without any restriction on the source of cells, the administration dose, and the site of transplantation; (3) comparison: saline, phosphate buffer saline (PBS), or no treatment; (4) outcome measure: cerebral infarct volume, apoptosis rate, neurological impairment scores, brain water content, Caspase-3, tumor necrosis factor *α* (TNF-*α*), interleukin 1*β* (IL-1*β*), and interleukin 6 (IL-6). Articles meeting any of the following criteria were excluded: (1) duplicate literature; (2) reviews, conference abstracts, editorials, letters to editors, case reports, and other meta-analyses; (3) studies in humans, or in vitro studies; (4) other sEVs than derived from mesenchymal stem cells; (5) incorrect or incomplete literature data that could not be included in the statistical analysis; (6) No relevant outcomes reported.

### 2.3. Quality Assessment

The quality assessment of the studies included in the present research was independently performed by two researchers using SYRCLE ‘s risk of bias tool for animal studies [52] recommending ten items of evaluation, evaluation results with “Y”, “N” and “U” represent, respectively, low risk of bias, bias risk, and uncertain risk of bias. The disagreements between the 2 investigators were settled by means of discussion until an agreement was reached with the third investigator.

### 2.4. Data Extraction

Two independent authors extracted the following details from the included studies and made a data extraction sheet: (1) the name of the first author; (2) year of publication; (3) country; (4) animal species, sex, and weight; (5) kind of anesthetic; (6) the source of MSCs; (7) MSCs isolation method; (8) MSCs characterization method; (9) MSCs positive marker; (10) EVs isolation method; (11) EVs characterization method; (12) the diameter of EVs; (13) EVs positive marker; (14) model of cerebral I/R; (15) the information of treatment group, including therapeutic drug dosage, method of administration, duration of treatment, and the same information of control group; (16) time point of extracting brain tissue; (17) mean value and standard deviation of outcomes. Because some records' published data were only in graphical format, we made efforts to contact authors for further information. When the response was not received, the numerical values were measured from the graphs by GetData Graph Digitizer 2.26 software.

### 2.5. Statistical Analysis

The pooled analyses were carried out with RevMan 5.3 software. I^2^ statistics is calculated and reported to assess the degree of heterogeneity. A fixed-effects model (I^2^<50%) or a random-effects model (I^2^>50%) was used depending on the value of I^2^. Funnel plots were used to visually estimate publication bias. We calculated the standard mean difference (SMD) with 95% confidence intervals (CIs).

## 3. Results

### 3.1. Study Selection

**Figure 1:**
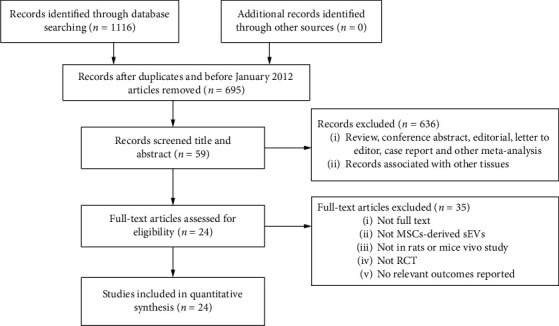
Flow chart of literature screening.

A total of 1116 articles were retrieved through the pertinent literature retrieval from the database, of which 421 were reduplicated or before January 2012 articles. After screening titles and abstracts, 636 were excluded because they were (1) reviews, conference abstracts, editorials, letters to the editor, case reports, and other meta-analyses and (2) records associated with other tissues. We then studied the remaining 59 full-text articles. Among them, 35 articles were excluded for at least one of the following reasons: (1) not full text; (2) not MSCs-derived sEVs; (3) not in rats or mice vivo study; (4) not RCT; (5) no relevant outcomes reported. Finally, 24 studies [26–49] were selected (Figure 1).

### 3.2. Characteristics of Included Studies

A cerebral I/R model for Balc/C mice was constructed in one of these research [32]， while Sprague-Dawley (SD) rats were the subjects of 16 experiments [26–31, 34, 35, 39, 40, 42, 44, 46–49], and C57BL/6 J mice were the subjects of 7 studies [33, 36–38, 41, 43, 45]. Only male animals were employed in 19 researches [26–31, 34, 35, 38–48]; other studies [32, 33, 36, 37, 49] did not report the sex of the animals. Anesthesia: 9 studies [26, 29, 30, 34, 35, 37, 38, 41, 47] used pentobarbital; 3 studies [28, 31, 48] used chloral hydrate; 1 study [36] used chloral hydrate and xylazine; 5 studies [27, 39, 40, 43, 46] used isoflurane; 1 study [32] used ketamine; 1 study [44] used ether; 1 study [45] used isoflurane oxygen/nitrous oxide mixture; 1 study [49] used uratan; and 2 studies [33, 42] not clearly named the anesthetics used. The middle cerebral artery occlusion (MCAO) model is used as I/R model in all studies. MSCs-derived sEVs were injected into the experimental group, the control group was injected with phosphate-buffered saline (PBS) or saline, 18 studies [27, 28, 30–38, 40, 43–48] via tail vein, 5 studies [26, 29, 41, 42, 49] via the lateral cerebral ventricle, and 1 study [39] via vein. The overall characteristics of included publications are shown in Table 1.

### 3.3. Isolation, Characterization, and Quantification of MSCs and EVs

MSCs were isolated via centrifugation alone (7 studies) [26, 33, 38, 39, 46, 48, 49]; by gradient centrifuge method (2 studies) [35, 43]; or by adherence method (2 studies) [30, 36]; or by centrifugation in combination with filtration methods (1 study) [40]; or by filtration methods (1 study) [28]. 11 studies [27, 29, 31, 32, 34, 37, 41, 42, 44, 45, 47] did not report the methods used to isolate MSCs. 16 studies (67%) characterized MSCs for MSCs positive markers [26, 28, 32–38, 41–46, 49]. MSCs were fixed and stained with Alizarin Red S for osteogenic differentiation and/or Oil Red O for adipogenic differentiation in 8 studies [28, 32, 33, 38, 41, 42, 44, 45] (Table 1).

EVs were isolated via ultracentrifugation (UC) alone (12 studies) [29, 31, 34, 35, 37, 38, 41–44, 46, 49]; by UC in combination with filtration methods and/or isolation kits (4 studies) [30, 32, 40, 47]; or by isolation kits (1 study) [45]; or by isolation kits in combination with low-speed centrifugation steps (1 study) [27]; or by sequential centrifugation (1 study) [36]; or by centrifugation (4 studies) [26, 28, 33, 48]. One study [39] did not report the methods used to isolate EVs. 23 studies [26, 28–49] (95.8%) characterized EVs using transmission electron microcopy (TEM) in combination with nanoparticle tracking analysis (NTA; 9 studies) [32–35, 38, 42–45] or dynamic light scattering analysis (DLS; 1 study) [41]. 24 studies [26–49] (100%) characterized EVs using western blot for protein markers in combination with flow cytometry (3 studies) [30, 36, 47] or bicinchoninic acid (BCA) protein (1 study) [32]. In addition, 22 studies [26, 28–38, 40–49] (91.7%) reported a range in EV size from 30–200 nm. 2 studies [27, 39] did not report the size of its EVs (Table 1).

### 3.4. Study Quality

The score of study quality ranged from three to nine in a total of ten points. Of which, 2 studies [32, 36] got three points; 12 studies [26–28, 30, 33–35, 37, 40, 41, 43, 49] got four points; 8 studies [29, 31, 39, 42, 44, 45, 47, 48] got five points; 1 study [38] got six points; and 1 study [46] got nine points. Most studies lacked reliable randomization methods, blinding methods, or allocation concealment. The methodological quality is concluded in Table 2.

### 3.5. Effectiveness

#### 3.5.1. Cerebral Infarction Volume

**Figure 2:**
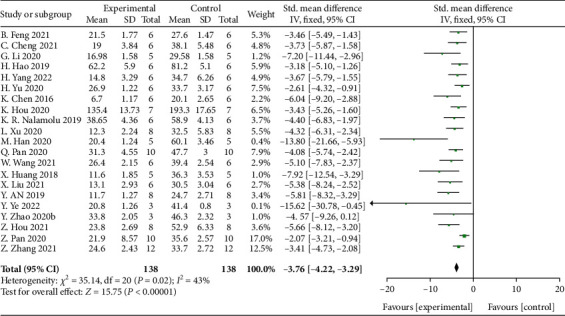
The forest plot: effects of MSCs-derived sEVs for decreasing the cerebral infarction size compared with control group.

Meta-analysis of 21 studies [26–29, 31–33, 35–41, 43–49] showed significant effects of MSCs-derived sEVs for decreasing the cerebral infarction volume compared with control group (*n* = 138, SMD: −3.76, 95% CI: −4.22 to −3.29, *P* < 0.00001; heterogeneity: *X*^2^ = 35.14, df = 20 (*P* = 0.02), *I*^2^ = 43%) (Figure 2).

#### 3.5.2. Apoptosis Rate

**Figure 3:**
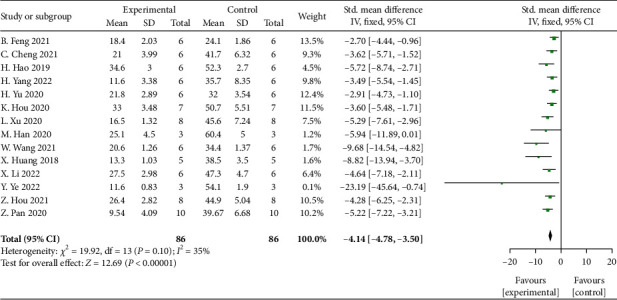
The forest plot: effects of MSCs-derived sEVs for decreasing apoptosis rate compared with control group.

Meta-analysis of 14 studies [26, 28, 29, 31, 33, 36, 38, 40–42, 44–46, 49] showed significant effects of MSCs-derived sEVs for decreasing the apoptosis rate compared with control group (*n* = 86, SMD: -4.14, 95% CI: −4.78 to −3.50, *P* < 0 00001; heterogeneity: *X*^2^ = 19.92, df = 13 (*P* = 0.10), *I*^2^ = 35%) (Figure 3).

#### 3.5.3. Neurological Impairment Score

**Figure 4:**
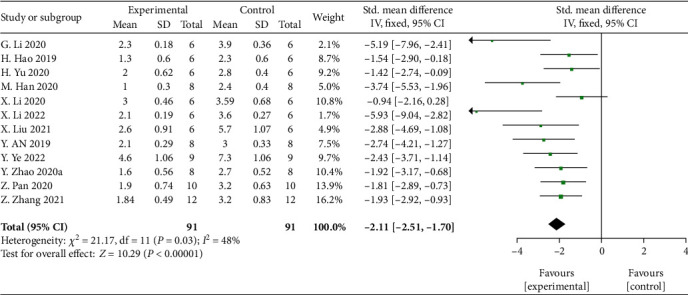
The forest plot: effects of MSCs-derived sEVs for decreasing the neurological impairment score compared with control group.

Meta-analysis of 12 studies [28, 30–32, 34, 35, 37, 40, 42, 46, 48, 49] showed significant effects of MSCs-derived exosomes for decreasing the neurological impairment score compared with control group (*n* = 91, SMD: -2.11, 95% CI: −2.51 to −1.70, *P* < 0 00001; heterogeneity: *X*^2^ = 21.17, df = 11 (*P* = 0.03), *I*^2^ = 48%) (Figure 4).

#### 3.5.4. Brain Water Content

**Figure 5:**
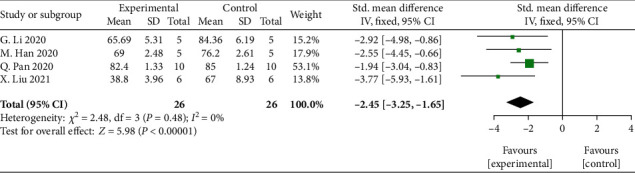
The forest plot: effects of MSCs-derived sEVs for decreasing the brain water content compared with control group.

Meta-analysis of 4 studies [32, 35, 40, 43] showed significant effects of MSCs-derived sEVs for decreasing the brain water content compared with control group (*n* = 26, SMD: −2.45, 95% CI: −3.25 to −1.65, *P* < 0.00001; heterogeneity: *X*^2^ = 2.48, df = 3 (*P* = 0.48), *I*^2^ = 0%) (Figure 5).

#### 3.5.5. Caspase-3

**Figure 6:**
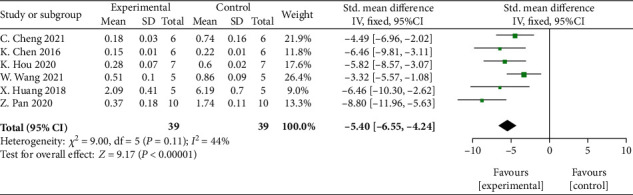
The forest plot: effects of MSCs-derived sEVs for reducing the level of caspase-3 compared with control group.

Meta-analysis of 6 studies [26, 29, 31, 36, 39, 44] showed significant effects of MSCs-derived sEVs for reducing the level of caspase-3 compared with control group (*n* = 39, SMD: −5.40, 95% CI: −6.55 to −4.24, *P* < 0.00001; heterogeneity: *X*^2^ = 9.00, df = 5 (*P* = 0.11), *I*^2^ = 44%) (Figure 6).

#### 3.5.6. TNF-*α*

**Figure 7:**
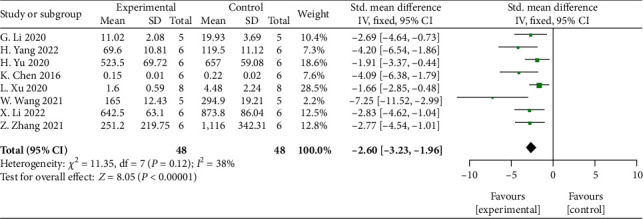
The forest plot: effects of MSCs-derived sEVs for reduced the expression of proinflammatory factor TNF-*α* compared with control group.

Meta-analysis of 8 studies [32, 33, 37, 39, 42, 44, 45, 49] showed significant effects of MSCs-derived sEVs for reducing the expression of proinflammatory factor TNF-*α* compared with control group (*n* = 48, SMD: −2.60, 95% CI: −3.23 to −1.96, *P* < 0.00001; heterogeneity: *X*^2^ = 11.35, df = 7 (*P* = 0.12), *I*^2^ = 38%) (Figure 7).

#### 3.5.7. IL-1*β*

**Figure 8:**
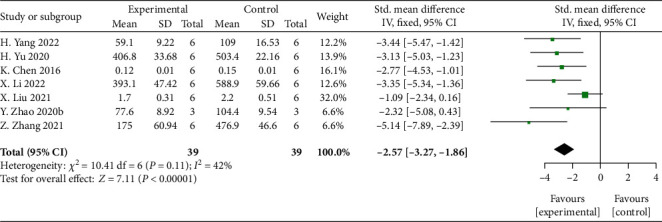
The forest plot: effects of MSCs-derived sEVs for reduced the expression of proinflammatory factor IL-1*β* compared with control group.

Meta-analysis of 7 studies [35, 37, 39, 42, 45, 47, 49] showed significant effects of MSCs-derived sEVs for reducing the expression of proinflammatory factor IL-1*β* compared with control group (*n* = 39, SMD: −2.57, 95% CI: −3.27 to −1.86, *P* < 0.00001; heterogeneity: *X*^2^ = 10.41, df = 6 (*P* = 0.11), *I*^2^ = 42)% (Figure 8).

#### 3.5.8. IL-6

**Figure 9:**
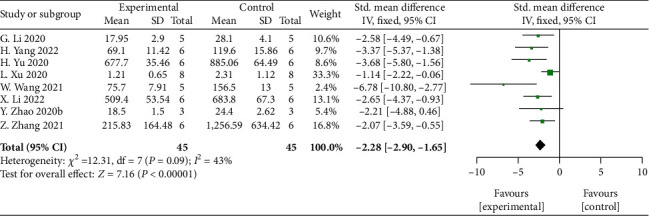
The forest plot: effects of MSCs-derived sEVs for reduced the expression of proinflammatory factor IL-6 compared with control group.

Meta-analysis of 8 studies [32, 33, 37, 42, 44, 45, 47, 49] showed significant effects of MSCs-derived sEVs for reducing the expression of proinflammatory factor IL-6 compared with control group (*n* = 45, SMD: −2.28, 95% CI: −2.90 to −1.65, *P* < 0.00001; heterogeneity: *X*^2^ = 12.31, df = 7 (*P* = 0.09), *I*^2^ = 43%) (Figure 9).

### 3.6. Publication Bias Analysis

**Figure 10:**
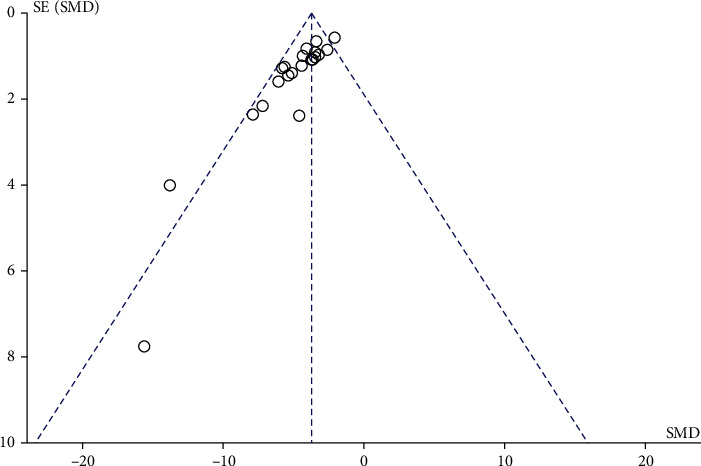
The funnel plot of the cerebral infarction volume in the included articles.

The publication bias was analyzed by funnel plot, and the volume of cerebral infarction was selected to draw the funnel plot. The funnel plot of cerebral infarction volume, as shown in Figure 10, is uneven in distribution and has a certain publication bias, which may be due to the inclination of positive publication and ignoring negative results and the lack of search of literature other than Chinese and English. Publication bias could only increase unreliability.

## 4. Discussion

Ischemic stroke remains a leading cause of mortality and disability worldwide, placing a huge economic burden on society. During ischemic cerebrovascular events, the most crucial goal for treatment is to restore blood flow to the ischemic penumbra. However, the restoration of blood flow will cause reperfusion injury, which eventually leads to neuronal death in ischemic penumbra via apoptosis and necrosis. Apoptosis is one of the major mechanisms of cell death during cerebral ischemia and reperfusion injury, which is the main cause of neuronal death in the central nervous system during cerebral ischemia [26]. Future focus could be directed towards inhibiting neuronal apoptosis to recover neuronal structure and function of rats after CIRI [31]. Additionally, inflammation also serves an important role during cerebral ischemia-reperfusion injury [28]. Despite efforts to reduce cerebral ischemia/reperfusion injury, an ideal therapeutic approach for clinical neuroprotection against ischemia/reperfusion injury is still lacking.

Previous studies suggested that cell-based therapy using MSCs may not only be an effective reparative treatment but also a brain-protective therapy that improves neurological recovery [53–55]. Recently, exosomes derived from MSCs have been found to carry various kinds of mediators, miRNAs, and proteins, which can mediate the function of MSCs [56–58]. There is growing evidence that MSCs-derived exosomes can play important roles in repairing brain-injured tissues [26]. However, we were unable to locate any systematic reviews or reviewers regarding the attenuation of CIRI by exosomes derived from MSCs in PUBMED or Web of Science. There are 24 control trials [26–49] published from 2016 to 2022 providing new evidence. Thus, an updated meta-analysis is essential. This meta-analysis is based on 24 controlled preclinical trials to demonstrate that MSCs-derived sEVs could significantly inhibit CIRI, in terms of cerebral infarct volume, apoptosis rate, neurological impairment scores, brain water content, and neuroinflammation.

Mesenchymal stem cells (MSCs) have been widely used in the experimental or clinical treatment of various ischemic diseases, but the therapeutic efficacy of MSCs on CIRI requires more research. The ethical issue is the main factor hindering advancement in clinical research. Human umbilical cord MSCs (hUMSCs), autologous adipose-derived MSCs, and autologous bone marrow-derived MCSs (BMSCs) are associated with minimal ethical controversy compared to other stem cells. Among the 24 studies included in this meta-analysis, the sources of MSCs were bone marrow MSCs in 12 studies [29, 30, 34–36, 38, 40, 42, 43, 45, 47, 49], human umbilical cord MSCs in 7 studies [27, 31, 32, 37, 44, 46, 48], and adipose MSCs in 5 studies [26, 28, 33, 39, 41]. Relative to human BMSCs, hUMSCs are more readily obtained, exhibit superior viability, are compatible with therapeutic methods featuring higher levels of patient acceptability and compliance, and are not susceptible to immune-mediated graft rejection [59, 60]. Stroke occurs frequently between the ages of 45 and 65, and there is an autologous bone marrow aging problem. In clinical research, it can minimize pain during bone marrow extraction and enhance volunteer compliance [37].

The sEVs derived from MSCs could mitigate nerve injury after cerebral I/R confirmed by some studies. Cheng et al. demonstrated that MSCs-derived exosomes attenuate ischemia-reperfusion brain injury and inhibit microglia apoptosis might via exosomal miR-26a-5p mediated suppression of CDK6 [36]. Furthermore, Li et al. drew a conclusion that exosomal miR-26b-5p could mitigate nerve injury after cerebral I/R by targeting CH25H and inactivating the TLR pathway [32]. Hou et al. also found that negative regulation of PTEN and activation of Akt mediated the effects of miR-29b-3p on the amelioration of brain injury caused by hypoxic ischemia [29]. Subsequently, they found out that miR-29b-3p delivered in exosomes from BMSCs accelerated angiogenesis of BMECs and hindered neuronal apoptosis after ischemic stroke via targeting PTEN and activating the Akt signaling pathway [29].

In the current analysis, the quality of included studies was considered as moderate, which ranged from three to nine out of a ten. The main drop points are that no study reported the allocation scheme concealment, whether the participants and the investigator adopted the blind method and whether the blind method was applied to the result evaluation. Secondly, only 2 studies [28, 46] (8.33%) reported the random allocation method. Therefore, future research should pay more attention to the application of the blind method in experimental design, and at the same time, the specific experimental implementation details should be reported comprehensively, so as to improve the repeatability and reliability of animal experimental results.

Various pharmacological agents have been shown to reduce CIRI in animal models. However, lack of neuroprotectant has been routinely utilized for clinical CIRI so far. One of the major results of this meta-analysis was that MSCs-derived sEVs significantly alleviated neurological impairment scores, reduced the volume of cerebral infarction and brain water content, and attenuated neuronal apoptosis in mice or rats MCAO model, and heterogeneity was not evident, indicating that sEVs showed consistent therapeutic potential in inhibiting CIRI and alleviating neuron damage. Furthermore, the main pathways of apoptosis include extracellular signal-triggered caspase activation and intracellular apoptotic enzyme release from mitochondria, which activate caspase [61]. As we can see, caspase plays an important role in apoptosis and is involved in the common pathway of various apoptotic signals. Among them, caspase-3 is the most important terminal cleavage enzyme in the process of cell apoptosis [62]. Our results provide evidence that MSCs-derived sEVs reduced the level of caspase-3 with no obvious heterogeneity observed, which confirmed that sEVs can suppress neuron apoptosis via caspase-3 pathway.

Acute ischemic stroke has been demonstrated to induce the inflammatory response accompanied by a significant increase in the expression levels of inflammatory and proinflammatory cytokines markers [63]. Microglia release proinflammatory cytokines such as IL-1*β*, IL-6, and TNF-*α* in the acute phase of ischemic stroke, impeding postinjury neural regeneration and producing poorer long-term neurological outcomes [64, 65]. Studies have proven that decreasing microglia-mediated neuroinflammation is beneficial during stroke recovery [65, 66]. The other of the major results of this meta-analysis was that MSCs-derived sEVs inhibited the expression of proinflammatory factors (TNF-*α*, IL-1*β*, IL-6) and attenuated microglia-mediated neuroinflammation after ischemic stroke and heterogeneity were not observed obviously. So, MSCs-derived sEVs can reduce CIRI by anti-inflammation.

Although results of this meta-analysis were supported by powerful proof, some limitations were worth noting. First, due to the relatively short number of trials, we were unable to conduct an in-depth metaregression analysis and subgroup analysis. Second, the neurological impairment scores included in this study varied over time, and the long-term follow-up effect could not be further analyzed, so that the long-term effect was not supported by corresponding evidence. Third, the parameters we chose are insufficient to demonstrate the full range of exosomes functions. Fourth, we acknowledge that the study did not retrieve unpublished literature and was limited to Chinese and English research, and the funnel chart suggests that there may be a certain publication bias, which could exaggerate the positive results. Fifth, most of the included studies did not report allocation concealment or blind method, which has a certain risk of bias. Sixth, because some data cannot be obtained directly in research, we measured the numerical values from the graphs, which led to possible deviations between estimated and actual statistical data. Finally, 24 studies included in this meta-analysis all used healthy adult rats or mice that fail to account for preexisting stroke risk factors such as hypertension, obesity, diabetes, sex, and aging. Generally, various factors exert important effects on the outcome in this stroke model, necessitating further research.

## 5. Conclusions

MSCs-derived sEVs could effectively attenuate CIRI in vivo and inhibit microglia-mediated neuroinflammation, which might be regarded as a novel insight for cerebral ischemic stroke. The preclinical results are encouraging for preparing and using feasibility studies in humans. However, more in-depth research is needed in the future to validate the therapeutic safety, in order to draw a more reliable and persuasive conclusion.

## Figures and Tables

**Table 1:** Characteristics of the 13 included studies.

1D	Study	Country	Animals	Anesthetic	The source of MSCs	MSCs isolation method	MSCs characterization method	MSCs positive marker	EVs isolation method	EVs characterization method	Diameter of EVs	EVs positive marker	Injury	Experimental group treatment	Control group treatment	Time point of extracting brain tissue	Route	Outcomes
1	B. Feng [38]	China	Male mice (C57BL/6 J, 22-25 g)	Sodium pentobarbital	Mice bone marrow	Centrifugation	Flow cytometry, alizarin red staining, oil red O staining, Alcian blue staining	CD29, CD44, SCA-1	Ultracentrifugation	TEM, NTA, western blot	30-120 nm	CD9, CD63, TSG101	MCA occlusion for 1 hour	200 *μ*g EVs	PBS	7 days after reperfusion	Via tail vein	Infarct volume, apoptosis
2	C. Cheng [36]	China	Adult mice (C57BL/6 J, 8 weeks, 250 g)	Chloral hydrate and xylazine	Mice bone marrow	Adherence method	Western blot	CD9, CD63, CD81, HSP70	Sequential centrifugation	TEM, western blot, flow cytometry	30–150 nm	CD9, CD63, CD81, HSP70,	MCA occlusion for 1 hour	200 *μ*L MSCs-EXOS	Saline	Not shown	Via tail vein	Infarct volume, apoptosis, Caspase-3
3	G. Li [32]	China	Wild mice (Balc/C, 4 weeks, 20 ± 5 g)	Ketamine	Human umbilical cord	Not stated	Oil red staining, alizarin-red staining	CD29, CD90, CD105	Ultracentrifugation, filtration	TEM, NTA, Western blot, BCA protein	120 nm	CD63, CD9, CD81, Alix	MCA occlusion	50 *μ*g/mL hUCMSCs-exos	PBS	24 hours after reperfusion	Via tail vein	Neurological deficit score, infarct size, water content, TNF-*α*，IL-6
4	H. Hao [28]	China	Adult male rats (SD, 8 weeks, 270–300 g)	Chloral hydrate	Rat adipose tissue	Filtration	Flow cytometry, alizarin red staining, oil red O staining	CD31	Centrifugation	TEM, western blot	30–100 nm	TSG101, HSP70	MCA occlusion for 2 hours	100 *μ*g of ADMSCs-exos	PBS	24 hours after reperfusion	Via tail vein	Neurological impairment scores, cerebral infarction volume, apoptosis
5	H. Yang [45]	China	Male mice (C57BL/6 J, 20-25 g)	Isoflurane oxygen/nitrous oxide mixture	Mice bone marrow	Not stated	Flow cytometry, alizarin red staining, oil red O staining, Alcian blue staining	CD44, CD90, CD29	Isolation kits	TEM, NTA, western blot	Around 110 nm	CD9, CD63, TSG101	MCA occlusion for 2 hours	10 *u*g exosomes	PBS	24 hours after reperfusion	Via tail vein	Infarct volume, apoptosis, TNF-*α*, IL-1*β*, IL-6
6	H. Yu [49]	China	Rats(8 weeks, SD, 250-270 g)	Uratan	Rat bone marrow	Centrifugation	Flow cytometry	CD29, CD90	Ultracentrifugation	TEM, western blot	40-130 nm	CD9, CD63, TSG101	MCA occlusion for 2 hours	100 *μ*g exosomes	PBS	24 hours after reperfusion	Via the lateral cerebral ventricle	Neurological deficit score, infarct volume, apoptosis, TNF-*α*, IL-1*β*, IL-6
7	K. Chen [39]	China	Male rats (SD, 350-375 g)	Inhalational isoflurane	Mini-pigs adipose tissues	Centrifugation	Flow cytometry	Not stated	Not stated	TEM, western blot	Not shown	Not stated	MCA occlusion for 50 min	100 *u*g exosomes	Not shown	60 days after reperfusion	Via vein	Infarct volume, Caspase-3, TNF-*α*, IL-1*β*
8	K. Hou [29]	China	Male rats (SD, 6–8 weeks, 250 ± 12 g)	Pentobarbital sodium	Rat bone marrow	Not stated	Not stated	Not stated	Ultracentrifugation	TEM, western blot	30-200 nm	CD80, CD63, TSG101	MCA occlusion	100 *μ*g/kg/d ^∗^ 3 days MSCs-Exo	Saline	72 hours after reperfusion	Via the lateral cerebral ventricle	Infarct volume, apoptotic level, Caspase-3
9	K. R. Nalamolu [27]	USA	Adult male rats (SD, 240 ± 20 g)	Isoflurane	Human umbilical cord blood	Not stated	Not stated	Not stated	Isolation kits, centrifugation	Western blot	Not shown	CD9, CD63	MCA occlusion for 2 hours	150 *μ*g exosomes	PBS	24 hours after reperfusion	Via tail vein	Infarct size
10	L. Xu [33]	China	Mice (C57BL/6, 25 ± 2 g)	Not shown	Mice adipose tissues	Centrifugation	Flow cytometry, oil red O staining	CD29, CD90, CD44, CD105	Centrifugation	TEM, NTA, western blot	30–100 nm	CD9, CD63, TSG101	MCA occlusion for 1 hour	Exosomes (400 *μ*g of protein)	Not shown	72 hours after reperfusion	Via tail vein	Infarct volume, cerebral apoptosis, TNF-*α*，IL-6
11	M. Han [40]	China	Adult male rats (SD, 7-8 weeks, 280–330 g)	Isoflurane	Rat bone marrow	Filtration, centrifugation	Not stated	Not stated	Ultracentrifugation, filtration	TEM, western blot	50–200 nm	CD9, TSG101	MCA occlusion for 2 hours	100 *μ*g MSCs-EVs	PBS	48 hours after reperfusion	Via tail vein	Neurological deficit score, infarct volume, water content, apoptosis
12	Q. Pan [43]	China	Male mice (C57BL/6, 6-8 weeks)	Isoflurane	Mice bone marrow	Gradient centrifuge method	Flow cytometry	CD34, CD45	Ultracentrifugation	TEM, NTA, western blot	100 ± 55 nm	CD63, TSG101	MCA occlusion for 2 hours	1 × 1010 particles MSC-Exs	PBS	48 hours after reperfusion	Via tail vein	Infarct volume, water content
13	W. Wang [44]	China	Male rats (SD, 280 ± 20 g)	Ether	Human umbilical cord	Not stated	Flow cytometry, alizarin red staining, oil red O staining	CD29, CD44, CD105	Ultracentrifugation	TEM, NTA, western blot	30–100 nm	CD9, CD63, Alix3	MCA occlusion for 90 min	100 *μ*g/day ^∗^ 3 days HMC-EV	PBS	72 hours after reperfusion	Via tail vein	Infarct volume, apoptosis, Caspase-3, TNF-*α*，IL-6
14	X. Huang [26]	China	Male rats (SD, 230–280 g)	Sodium pentobarbital	Adipose tissue from normal rats	Centrifugation	Immunofluorescence staining	CD29, CD90, CD44, CD105	Centrifugation	TEM, western blot	100 nm	CD63, CD81, TSG101	MCA occlusion for 60 min	100 *μ*g/kg/day ^∗^ 3 days exosomes	Not shown	72 hours after reperfusion	Via the lateral cerebral ventricle	Infarct volume, apoptotic, Caspase-3
15	X. Li [34]	China	Adult male rats(SD, 250-270 g)	Sodium pentobarbital	Bone marrow cavity of rats	Not stated	Flow cytometry	CD90, CD44, CD105	Ultracentrifugation	TEM, NTA, western blot	Around 100 nm	CD9, TSG101, Alix	MCA occlusion for 90 min	100 *μ*g exosomes	PBS	72 hours after reperfusion	Via tail vein	mNSS score
16	X. Li [42]	China	Male rats (SD, 260–280 g)	Not shown	Rat bone marrow	Not stated	Flow cytometry, alizarin red staining, oil red O staining	CD29, D54, CD90	Ultracentrifugation	TEM, NTA, western blot	30–150 nm	CD63, CD9, CD81	MCA occlusion for 2 hours	100 *μ*g/kg exosomes	Not shown	72 hours after reperfusion	Via the lateral cerebral ventricle	Neurological function score, apoptosis, TNF-*α*, IL-1*β*, IL-6
17	X. Liu [35]	China	Adult male rats (SD, 280–300 g)	Pentobarbital	Rat bone marrow	Gradient centrifuge method	Flow cytometry	CD29, CD90	Ultracentrifugation	TEM, NTA, western blot	30–150 nm	CD9, TSG101	MCA occlusion for 2 hours	120 *μ*g of BMSCs-exos in 2 mL PBS	PBS	24 hours after reperfusion	Via tail vein	Neurological impairment scores, brain water content, cerebral infarction volume, IL-1*β*
18	Y. An [48]	China	Male rats (SD, 240–300 g)	Chloral hydrate	Human umbilical cord	Centrifugation	Not stated	Not stated	Centrifugation	TEM, western blot	30-100 nm	CD63, TSG101	MCA occlusion for 2 hours	Not shown	PBS	14 days after reperfusion	Via tail vein	Neurological deficit score, infarct volume
19	Y. Ye [46]	China	Male rats (SD, 8 weeks, 250 ± 30 g	Isofurane	Human umbilical cord	Centrifugation	Not stated	CD90, CD105	Ultracentrifugation	TEM, western blot	50-120 nm	CD9, CD63	MCA occlusion for 2 hours	80 *μ*g ^∗^ 3 days exosomes	PBS	48 hours after reperfusion	Via tail vein	Neurological deficit score, infarct volume, apoptosis
20	Y. Zhao [30]	China	Male rats (SD, 270 ± 10 g)	Pentobarbital sodium	Rat bone marrow	Adherence method	Not stated	Not stated	Ultracentrifugation, filtration	TEM, western blot, flow cytometry	30-150 nm	CD63, CD81	MCA occlusion for 90 min	200 *μ*L MSC-exos	Saline	7 days after reperfusion	Via tail vein	Neurological severity scores (NSS)
21	Y. Zhao [47]	China	Male rats (SD, 260-280 g)	Pentobarbital sodium	Rat bone marrow	Not stated	Not stated	Not stated	Ultracentrifugation, filtration	TEM, western blot, flow cytometry	30-150 nm	CD63, CD81	MCA occlusion for 90 min	200 *μ*L MSC-exos	Normal saline	28 days after reperfusion	Via tail vein	Infarct volume, IL-1*β*, IL-6
22	Z. Hou [41]	China	Male mice (C57BL/6, 8 weeks)	Sodium pentobarbital	Mice white adipose tissues	Not stated	Flow cytometry, alkaline phosphatase staining, oil red O staining, Alcian blue staining	CD29, CD44, CD73, CD90, CD105, CD166	Ultracentrifugation	TEM, DLS, western blot	Around 100 nm	CD63, TSG101	MCA occlusion for 1 hour	100 mmol/kg/d ^∗^ 3 days EVs	PBS	72 hours after reperfusion	Via the lateral cerebral ventricle	Infarct volume, apoptosis
23	Z. Pan [31]	China	Adult male rats (SD, 280–300 g)	Chloral hydrate	Human umbilical cord	Not stated	Not stated	Not stated	Ultracentrifugation	TEM, western blot	30–100 nm	CD9, CD63, CD81, TSG101	MCA occlusion for 2 hours	100 *u*g exosomes	Saline	24 hours after reperfusion	Via tail vein	Neurological impairment scores, cerebral infarction volume, apoptosis, Caspase-3
24	Z. Zhang [37]	China	Mice (C57BL/6, 8 weeks, 20-30 g)	Sodium pentobarbital	Human umbilical cord	Not stated	Flow cytometry	CD73, CD105, CD90	Ultracentrifugation	TEM, western blot	30-150 nm	CD9, Alix, TSG101	MCA occlusion for 1 hour	50 *μ*g exosomes	PBS	72 hours after reperfusion	Via tail vein	Neurological function scores, infarct volume, TNF-*α*, IL-1*β*, IL-6

**Table 2:** Risk of bias of the included studies.

ID	Study	①	②	③	④	⑤	⑥	⑦	⑧	⑨	⑩	Score
1	B. Feng [38]	U	Y	U	Y	Y	U	U	Y	Y	Y	6
2	C. Cheng [36]	U	U	U	U	U	U	U	Y	Y	Y	3
3	G. Li [32]	U	U	U	Y	U	U	U	U	Y	Y	3
4	H. Hao [28]	Y	Y	U	U	U	U	U	Y	Y	U	4
5	H. Yang [45]	U	Y	U	Y	U	U	U	Y	Y	Y	5
6	H. Yu [49]	U	Y	U	Y	U	U	U	U	Y	Y	4
7	K. Chen [39]	U	Y	U	Y	U	U	U	Y	Y	Y	5
8	K. Hou [29]	U	Y	U	Y	U	U	U	Y	Y	Y	5
9	K. R. Nalamolu [27]	U	Y	U	Y	U	U	U	Y	Y	U	4
10	L. Xu [33]	U	U	U	Y	U	U	U	Y	Y	Y	4
11	M. Han [40]	U	Y	U	Y	U	U	U	U	Y	Y	4
12	Q. Pan [43]	U	Y	U	Y	U	U	U	U	Y	Y	4
13	W. Wang [44]	U	Y	U	Y	U	U	U	Y	Y	Y	5
14	X. Huang [26]	U	Y	U	Y	U	U	U	U	Y	Y	4
15	X. Li [34]	U	Y	U	U	U	U	U	Y	Y	Y	4
16	X. Li [42]	U	Y	U	Y	U	U	Y	U	Y	Y	5
17	X. Liu [35]	U	Y	U	Y	U	U	U	U	Y	Y	4
18	Y. AN [48]	U	Y	U	Y	U	U	U	Y	Y	Y	5
19	Y. Ye [46]	Y	Y	Y	Y	Y	U	Y	Y	Y	Y	9
20	Y. Zhao [30]	U	Y	U	Y	U	U	U	U	Y	Y	4
21	Y. Zhao [47]	U	Y	U	Y	U	U	U	Y	Y	Y	5
22	Z. Hou [41]	U	Y	U	Y	U	U	U	U	Y	Y	4
23	Z. Pan [31]	U	Y	U	Y	U	U	U	Y	Y	Y	5
24	Z. Zhang [37]	U	U	U	Y	U	U	Y	U	Y	Y	4

①: Was the allocation sequence adequately generated and applied; ②: were the groups similar at baseline or were they adjusted for confounders in the analysis; ③: was the allocation adequately concealed; ④: were the animals randomly housed during the experiment; ⑤: were the caregivers and/or investigators blinded from knowledge which intervention each animal received during the experiment; ⑥: were animals selected at random for outcome assessment; ⑦: was the outcome assessor blinded; ⑧: were incomplete outcome data adequately addressed; ⑨: are reports of the study free of selective outcome reporting; ⑩: was the study apparently free of other problems that could result in high risk of bias. Y: yes; N: no; U: uncertain.

## Data Availability

The raw data supporting the conclusions of this article will be made available by the authors, without undue reservation.

## Supplementary Materials

Supplementary Materials File S1: PRISMA checklist for this systematic review. File S2: Database retrieval strategy of PubMed.
