# Supplementary material for: Inhibition of Cerebral Ischemia/Reperfusion Injury by MSCs-Derived Small Extracellular Vesicles in Rodent Models: A Systematic Review and Meta-Analysis
Source: Neural Plast. 2022 Oct 6;2022:3933252. doi: 10.1155/2022/3933252 (PMC9633211; doi:10.1155/2022/3933252)
Supplement: Supplementary Materials — File S1: PRISMA checklist for this systematic review. File S2: Database retrieval strategy of PubMed. [file 3933252.f1.zip › Supplementary 2.docx]

**Supplementary 2: Database retrieval strategy of PubMed**

1. **"Extracellular Vesicles"[Mesh]**
2. **(((((((((EVs[Title/Abstract]) OR (Extracellular Vesicle[Title/Abstract])) OR (Vesicle, Extracellular[Title/Abstract])) OR (Vesicles, Extracellular[Title/Abstract])) OR (Exovesicles[Title/Abstract])) OR (Exovesicle[Title/Abstract])) OR (Apoptotic Bodies[Title/Abstract])) OR (Apoptotic Body[Title/Abstract])) OR (Bodies, Apoptotic[Title/Abstract])) OR (Body, Apoptotic[Title/Abstract])**
3. #1 OR #2
4. **"Exosomes"[Mesh]**
5. **(((Endosomes[Title/Abstract]) OR (Secretory Vesicles[Title/Abstract])) OR (Cell-Derived Microparticles[Title/Abstract])) OR (Exosome Multienzyme Ribonuclease Complex[Title/Abstract])**
6. #4 OR #5
7. #3 OR #6
8. "Mesenchymal Stem Cells"[Mesh]
9. ((((((((((((((((((((((((((((((((((((((((((Stem Cell, Mesenchymal[Title/Abstract]) OR (Mesenchymal Stem Cell[Title/Abstract])) OR (Stem Cells, Mesenchymal[Title/Abstract])) OR (Bone Marrow Mesenchymal Stem Cells[Title/Abstract])) OR (Bone Marrow Mesenchymal Stem Cell[Title/Abstract])) OR (Bone Marrow Stromal Cells[Title/Abstract])) OR (Bone Marrow Stromal Cell[Title/Abstract])) OR (Bone Marrow Stromal Cells, Multipotent[Title/Abstract])) OR (Multipotent Bone Marrow Stromal Cell[Title/Abstract])) OR (Multipotent Bone Marrow Stromal Cells[Title/Abstract])) OR (Adipose-Derived Mesenchymal Stem Cells[Title/Abstract])) OR (Adipose Derived Mesenchymal Stem Cells[Title/Abstract])) OR (Adipose-Derived Mesenchymal Stromal Cells[Title/Abstract])) OR (Adipose Derived Mesenchymal Stromal Cells[Title/Abstract])) OR (Mesenchymal Stem Cells, Adipose-Derived[Title/Abstract])) OR (Mesenchymal Stem Cells, Adipose Derived[Title/Abstract])) OR (Adipose-Derived Mesenchymal Stem Cell[Title/Abstract])) OR (Adipose Derived Mesenchymal Stem Cell[Title/Abstract])) OR (Adipose Tissue-Derived Mesenchymal Stem Cell[Title/Abstract])) OR (Adipose Tissue Derived Mesenchymal Stem Cell[Title/Abstract])) OR (Adipose Tissue-Derived Mesenchymal Stem Cells[Title/Abstract])) OR (Adipose Tissue Derived Mesenchymal Stem Cells[Title/Abstract])) OR (Adipose Tissue-Derived Mesenchymal Stromal Cells[Title/Abstract])) OR (Adipose Tissue Derived Mesenchymal Stromal Cells[Title/Abstract])) OR (Adipose Tissue-Derived Mesenchymal Stromal Cell[Title/Abstract])) OR (Adipose Tissue Derived Mesenchymal Stromal Cell[Title/Abstract])) OR (Mesenchymal Stromal Cells[Title/Abstract])) OR (Mesenchymal Stromal Cell[Title/Abstract])) OR (Stromal Cell, Mesenchymal[Title/Abstract])) OR (Stromal Cells, Mesenchymal[Title/Abstract])) OR (Multipotent Mesenchymal Stromal Cells[Title/Abstract])) OR (Multipotent Mesenchymal Stromal Cell[Title/Abstract])) OR (Mesenchymal Stromal Cells, Multipotent[Title/Abstract])) OR (Mesenchymal Progenitor Cell[Title/Abstract])) OR (Mesenchymal Progenitor Cells[Title/Abstract])) OR (Progenitor Cell, Mesenchymal[Title/Abstract])) OR (Progenitor Cells, Mesenchymal[Title/Abstract])) OR (Wharton Jelly Cells[Title/Abstract])) OR (Wharton's Jelly Cells[Title/Abstract])) OR (Wharton's Jelly Cell[Title/Abstract])) OR (Whartons Jelly Cells[Title/Abstract])) OR (Bone Marrow Stromal Stem Cells[Title/Abstract])) OR (MSCs[Title/Abstract])
10. #8 OR #9
11. "Reperfusion Injury"[Mesh]
12. (((((((((Reperfusion Injuries[Title/Abstract]) OR (Reperfusion Damage[Title/Abstract])) OR (Damage, Reperfusion[Title/Abstract])) OR (Reperfusion Damages[Title/Abstract])) OR (Ischemia-Reperfusion Injury[Title/Abstract])) OR (Ischemia Reperfusion Injury[Title/Abstract])) OR (Injury, Ischemia-Reperfusion[Title/Abstract])) OR (Injury, Ischemia Reperfusion[Title/Abstract])) OR (Ischemia-Reperfusion Injuries[Title/Abstract])) OR (Injury, Reperfusion[Title/Abstract])
13. #11 OR #12
14. #10 AND #13
15. #14 AND #7
